# First Year Real Life Experience With Intravitreal Brolucizumab for Treatment of Refractory Neovascular Age-Related Macular Degeneration

**DOI:** 10.3389/fphar.2022.860784

**Published:** 2022-05-30

**Authors:** Alaa Din Abdin, Wissam Aljundi, Khalil El Jawhari, Shady Suffo, Isabel Weinstein, Berthold Seitz

**Affiliations:** Department of Ophthalmology, Saarland University Medical Center UKS, Homburg, Germany

**Keywords:** neovascular age-related macular degeneration (nAMD), intraocular inflammation, intravitreal brolucizumab, switching therapy, refractory macular edema

## Abstract

**Purpose:** To assess the morphological and functional outcomes within the first year of treatment with intravitreal brolucizumab for refractory neovascular age-related macular degeneration (nAMD).

**Methods:** This retrospective study included 21 eyes from 19 patients with refractory nAMD followed for 12 months. All patients were switched to brolucizumab after treatment with at least two other anti-vascular endothelial growth factors (VEGF). All eyes received 3x brolucizumab 6 mg/0.05 ml intravitreal injections (IVI) monthly as an upload phase. Then eyes received an IVI every 8 weeks with interval adjustment to every 12 weeks if disease activity was not present. Main outcome measures: best corrected visual acuity (BCVA), central macular thickness (CMT) and retinal fluid distribution. In addition, we reported the adverse event rate.

**Results:** The number of previous anti-VEGF IVIs/eye was 36 ± 22 before switching to brolucizumab. BCVA (ETDRS) was 51 ± 16 before treatment and 50 ± 19 at week 52 (*p* = 0.6). CMT was 374 ± 158 μm before treatment and 298 ± 92 μm at week 52 (*p* = 0.01). The number of IVIs/eye decreased from 9.6 ± 1.9 IVIs in the last year before switching to 6.4 ± 0.9 IVIs in the first year after switching to brolucizumab (*p* < 0.001). The rate of eyes with subretinal fluid and pigment epithelial detachment decreased at week 52. Finally, two cases of intraocular inflammation were observed as adverse events.

**Conclusion:** In the first year of treatment, intravitreal brolucizumab was able to stabilize visual acuity with significantly less IVIs in patients with refractory nAMD. It also improved anatomic outcomes in these patients, particularly reducing subretinal fluid and pigment epithelial detachment and subsequently central macular thickness. However, two cases of intraocular inflammation were observed as adverse events.

## Introduction

Neovascular age-related macular degeneration (nAMD) is an accelerating disease that represents an increasingly high burden for both patients and health care systems in developed countries (Jonas et al., 2017).

The important role of vascular endothelial growth factor (VEGF) in the development of macular neovascularization (MNV) related to nAMD has been confirmed (Augustin and Kirchhof, 2014). Indeed, it rapidly triggers angiogenesis, enhances vascular permeability, takes part in the disintegration of the blood-retinal barrier, and facilitates an inflammatory reaction (Ferrara et al., 2003). Therefore, intravitreal injections (IVI) of anti-VEGF agents would currently be considered as the gold standard therapy for nAMD, with a primary target of improving or preserving visual acuity (Li et al., 2020).

At present, three anti-VEGF agents have been licensed in Europe and the United States to treat nAMD: ranibiziumab, aflibercept and brolucizumab (Markham, 2019; Li et al., 2020).

Brolucizumab (Beovu^®^, Novartis Pharma GmbH, Nuernberg, Germany) was approved by regulatory authorities in the United States in October 2019 and the European Union in February 2020 for the treatment of nAMD. It is a low molecular weight (26 kDa) single-chain antibody fragment with high affinity against all forms of VEGF-A, better tissue penetration and higher molar concentration (Holz et al., 2016; Dugel et al., 2020; Dugel et al., 2021).

The clinical trial data from HAWK and HARRIER indicate that brolucizumab has comparable best-corrected visual acuity compared to aflibercept, with better anatomic outcomes. There is an opportunity to extend the dosing regimen to 12-week intervals, which could relieve the treatment burden (Dugel et al., 2021).

On the other hand, the HAWK and HARRIER studies showed a comparable safety profile of brolucizumab and aflibercept, except for a higher rate of intraocular inflammatory events (4.4%) in eyes being treated with 6 mg brolucizumab compared with aflibercept (0.8%) (Dugel et al., 2021). In addition, some clinical studies have reported intraocular inflammatory events with retinal vasculitis and retinal vascular occlusion with occasional severe vision loss after treatment with brolucizumab (Baumal et al., 2021; Holz et al., 2021).

The purpose of the present study was to assess the morphologic and functional outcomes and adverse effects within the first year of treatment with intravitreal brolucizumab for refractory macular edema due to nAMD.

## Materials and Methods

This retrospective monocentral study included 21 eyes of 19 patients with refractory macular edema due to nAMD followed for 12 months.

Refractory macular edema was defined morphologically as persistent intraretinal fluid (IRF) and/or subretinal fluid (SRF) and/or retinal pigment epithelial detachment (PED) despite treatment with at least two anti-VEGFs. This was identified using spectral-domain optical coherence tomography (SD-OCT).

All patients were switched to brolucizumab after treatment with at least two other anti-VEGFs, including ranibizumab, aflibercept and bevacizumab. All eyes received 3x brolucizumab 6 mg/0.05 ml IVIs monthly for an upload phase. Then, eyes received an IVI every 8 weeks with interval adjustment to every 12 weeks if disease activity was not present (Dugel et al., 2021).

All IVIs were performed between 16 March 2020, and 31 December 2021, at a designated center for intravitreal injections in our Department of Ophthalmology at Saarland University Medical Center (Abdin et al., 2020).

The inclusion criteria were.

1) Eyes with refractory macular edema due to nAMD (as defined above)
2) A minimum follow-up of 12 months.

The exclusion criteria were.

1) History of treatments with photodynamic therapy (PDT).
2) Eyes with macular scarring preventing a change in visual function.
3) Eyes with coexisting vitreoretinal pathology.
4) Intraocular surgery (cataract surgery, pars plana vitrectomy) within the first year of treatment with brolucizumab.

Naïve patients were not included in this study.

Main outcome measures included:

• Best corrected visual acuity (BCVA) as measured on a Snellen decimal scale and converted to approximate ETDRS (Early Treatment Diabetic Retinopathy Study) letter scores (Beck et al., 2003).
• Central macular thickness (CMT) as measured by (Spectralis SD-OCT; Heidelberg Engineering, Heidelberg, Germany) and defined as the mean retinal thickness (µm) between the internal limiting membrane (ILM) and the basement membrane of Bruch (BM) in the central 1 mm of the fovea.
• The number of IVIs before and during the first year of treatment.
• The presence of IRF, SRF, and PED was detected in OCT at baseline and each follow-up visit.

All outcomes were assessed at baseline, then at week 4, 8, 16, 20, 28, 36, 44 and 52 after treatment. The day of the first brolucizumab IVI was considered as the baseline follow-up.

In addition, all patients were followed up 2–5 days after each IVI according to the guidelines of the German Society of Ophthalmology (German Society of Ophthalmology et al., 2021). At each visit, a complete ophthalmic examination, including slit-lamp examination and funduscopy after pupil dilation, was performed to detect any signs of intraocular inflammation (IOI) including retinal vasculitis. Furthermore, patients were educated about the symptoms of IOI and advised to report immediately if any ocular or systemic adverse events were noted.

### Statistical Analysis

Statistical analysis was performed using SPSS (IBM SPSS Statistics V.26). A Mann-Whitney *U* test (nonparametric statistics) was performed to examine the effect of time on BCVA, CMT, and the number of injections between each follow-up visit and baseline. A Chi-square test was used to compare the difference in retinal fluid rates before and after treatment. Data was presented as mean ± standard deviation (95% CI). Results were considered statistically significant if the *p* value was <0.05.

## Results

The patients’ baseline characteristics are summarized in Table 1.

**TABLE 1:** Baseline characteristics of the study group (mean ± SD).

Age (years)	76 ± 8
Gender (Male:Female)	29%:71%
Eye (Right:Left)	48%:52%
Previous IVIs	36 ± 22
MNV type (1:2)	62%:38%
BCVA (ETDRS)	51 ± 16
CMT (µm)	374 ± 158

IVI, intravitreal injection; MNV, Macular neovascularization; BCVA, Best corrected visual acuity; ETDRS, Early Treatment of Diabetic Retinopathy Study; CMT, Central macular thickness

**FIGURE 1:**
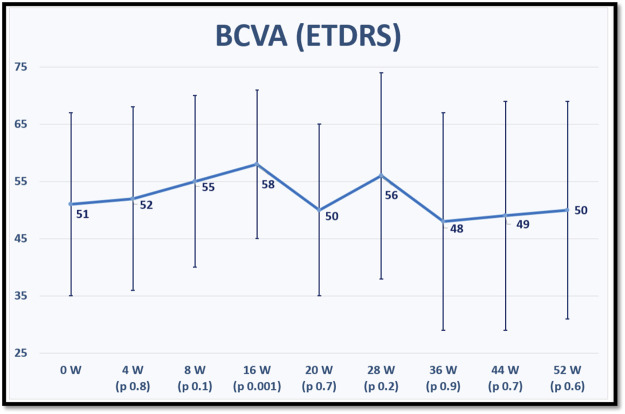
shows the mean best corrected visual acuity (BCVA) in Early Treatment of Diabetic Retinopathy Study (ETDRS) letter score at baseline and for each fellow up visit after switching to brolucizumab. *p* values refer to statistical differences between each time points and baseline.

The mean BCVA for each time point is displayed in Figure 1.

**FIGURE 2:**
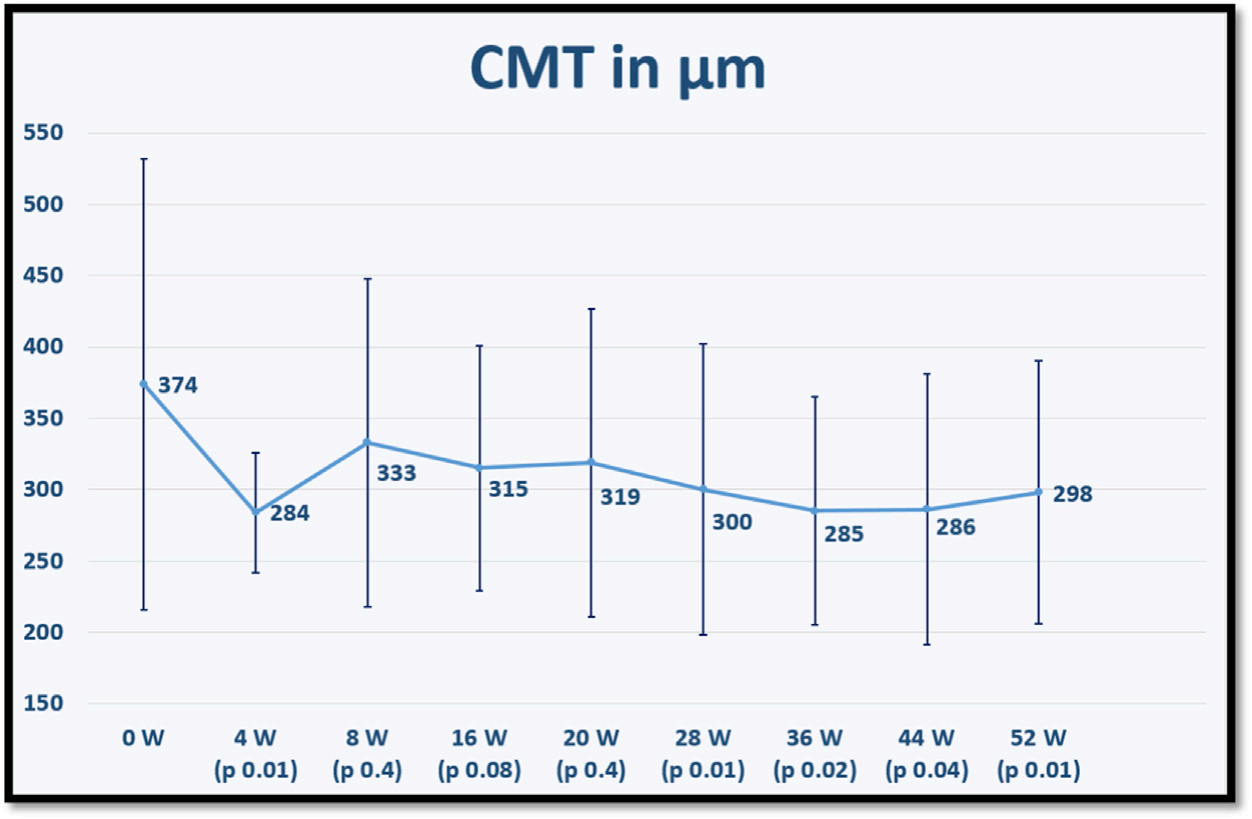
shows the mean central macular thickness (CMT) at baseline and for each fellow up visit after switching to brolucizumab. *p* values refer to statistical differences between each time points and baseline.

The mean CMT for each time point is displayed in Figure 2.

**FIGURE 3:**
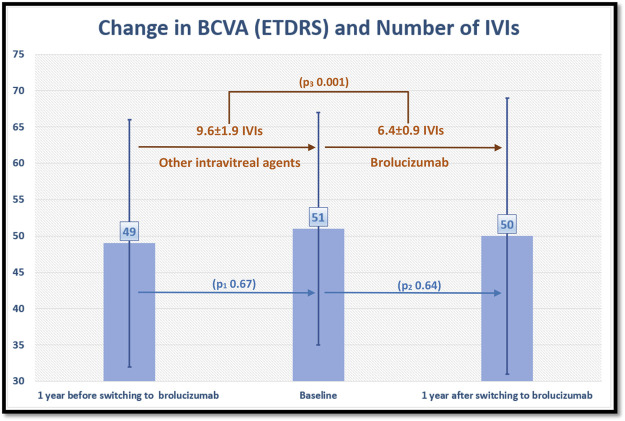
shows the change in best corrected visual acuity (BCVA) in Early Treatment of Diabetic Retinopathy Study (ETDRS) letter score and number of intravitreal injections (IVIs) per eye 1 year before and after switching to brolucizumab. The number of IVIs/eye decreased after switching to brolucizumab, whereas BCVA remained unchanged. P_1, 2_ values refer to statistical differences in BCVA between each time points. P_3_ value refers to the statistical difference in the number of IVIs between time points.

The number of previous anti-VEGF IVIs was 36 ± 22 before switching to brolucizumab (ranibizumab 9.6 ± 9.2 IVIs, aflibercept 22.7 ± 17.5 and bevacizumab 6.9 ± 8.2 IVIs). The number of IVIs/eye before and after switching to brolucizumab is displayed in Figure 3.

**FIGURE 4:**
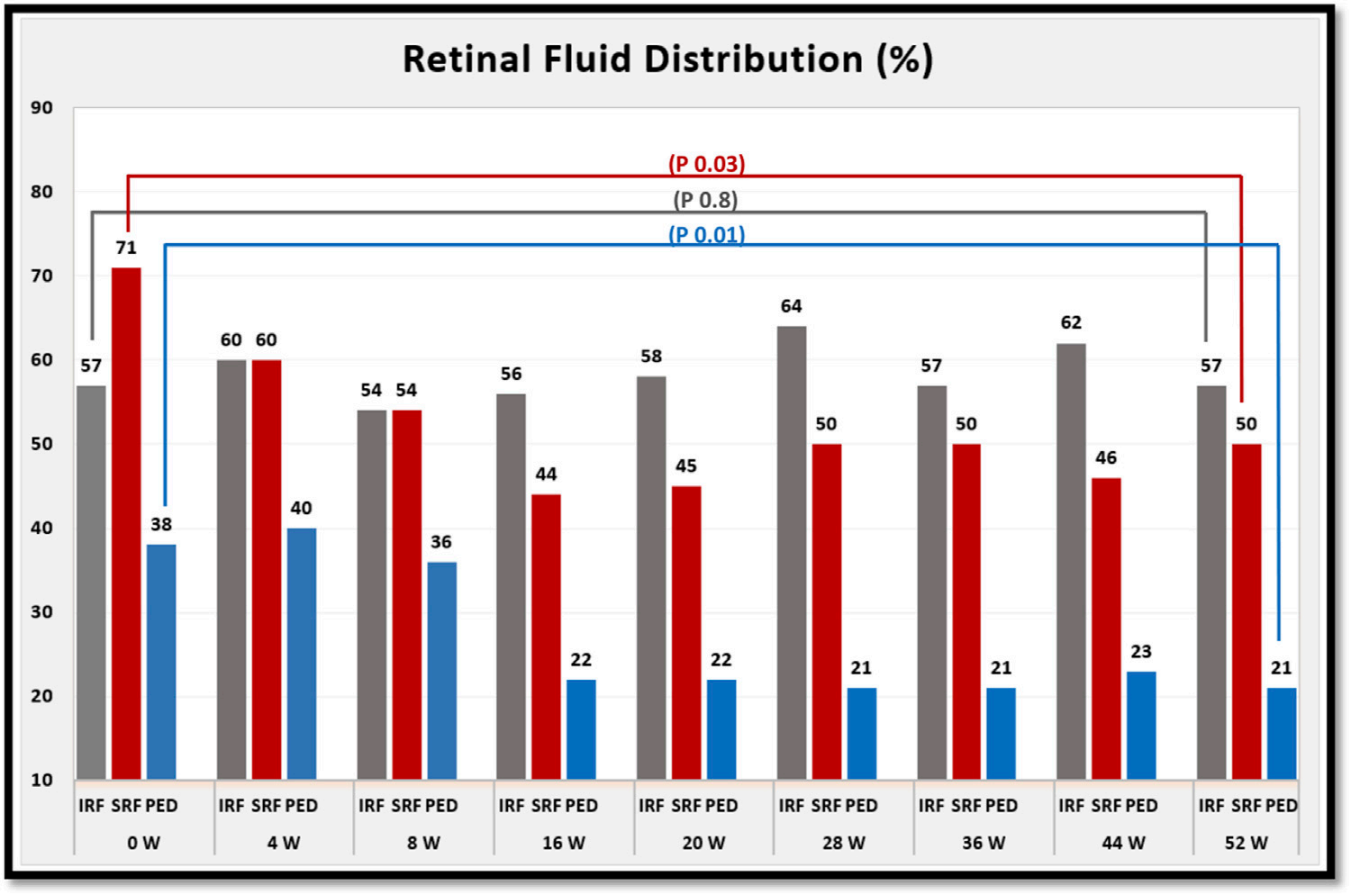
shows the percentage of eyes with each type of retinal fluid at baseline and for each follow-up visit after switching to brolucizumab. At week 52 of treatment, the percentage of eyes with subretinal fluid (SRF) and pigment epithelial detachment (PED) decreased. However, the percentage of eyes with intraretinal fluid (IRF) was comparable between baseline and week 52 of treatment. *p* values refer to the statistical differences between baseline and week 52 of treatment.

The percentage of eyes with each type of retinal fluid at each time point are shown in Figure 4.

A completely dry macula was observed in seven eyes (33.3%) during the treatment period, two at week 4, one at week 8, one at week 16, one at week 20, one at week 24 and one at week 44. Consequently, the treatment interval was extended to 12 weeks in these eyes. However, recurrence was observed in six eyes after 8.8 ± 3.3 weeks and the treatment interval was again shortened to 8 weeks.

### Adverse Events

In four eyes, treatment with brolucizumab was discontinued because of observed adverse effects (Table 2).

**TABLE 2:** Observed adverse events.

Adverse event	Gender	Number of patients	Week of incidence
Anterior uveitis	F	1	16
Intermediate uveitis	F	1	8
Ocular hypertension	F	1	16
Cerebrovascular accident	M	1	33

An 85-year-old woman who had previously received 25 IVIs of aflibercept and 7 IVIs of ranibizumab was switched to brolucizumab for refractory IRF and SRF. She received a total of 3 IVIs of brolucizumab. 8 weeks after the third brolucizumab-IVI, she presented with a headache. Clinical examination revealed an increase in intraocular pressure (IOP) to 50 mmHg, with a normal depth anterior chamber without signs of IOI. The optic disc was within normal limits, and the cup/disc ratio was 0.3. She had no history of glaucoma. The patient was treated with systemic intravenous acetazolamide (500 mg) and local combined eye drops “dorzolamide (hydrochloride) 2% + timolol (maleate) 0.5%” twice daily. IOP improved to 23 mmHg within 3 h. She continued local therapy for 4 weeks. This acute ocular hypertension was probably not due to brolucizumab, but treatment was discontinued as a precaution and later resumed with a previously used anti-VEGF agent.

A 79-year-old man who had previously received 3 IVIs of aflibercept and 17 IVIs of ranibizumab was switched to brolucizumab due to refractory IRF. He received a total of six IVIs of brolucizumab. 1 week after the sixth brolucizumab-IVI, he developed a CVA that was probably not attributable to brolucizumab, but treatment was terminated as a precaution and later restarted with a previously used anti-VEGF agent.

### Intraocular Inflammation (IOI) Was Observed in Two Eyes of Two Patients

Patient 1: A 78-year-old woman who had previously received 15 IVIs of aflibercept and 13 IVIs of ranibizumab was switched to brolucizumab due to refractory PED and SRF. She received a total of 2 IVIs of brolucizumab. 3 days after the second brolucizumab-IVI, she reported worsening visual acuity with ocular pain. Clinical examination revealed a decrease in BCVA from 20/100 to 20/200 with intermediate uveitis (vitreous cells +2, vitreous opacity +2), fundus examination revealed normal retinal findings with no evidence of retinal vasculitis or retinal vascular occlusions. An immediate pars plana vitrectomy with intravitreal antibiotics was performed for suspected endophthalmitis. A vitreous biopsy was performed in order to identify a potentially infectious cause of endophthalmitis. However, a PCR essay was found to be negative. The patient was treated with high-frequency (initially hourly) administration of prednisolone acetate eye drops (1%) and systemic prednisolone (1 mg/kg body weight) for 3 days and then tapered off over a period of 2 weeks. Ocular inflammation improved within 2 weeks, while visual acuity recovered to 20/80 after 1 month. Treatment with brolucizumab was discontinued and the patient was switched back to a previously used anti-VEGF agent with no further adverse events.

Patient 2: A 70-year-old woman who had previously received 3 IVIs of aflibercept and 10 IVIs of ranibizumab was switched to brolucizumab due to refractory IRF. She received a total of 4 IVIs of brolucizumab. 2 days after the fourth brolucizumab-IVI, she reported worsening visual acuity with eye redness. Clinical examination showed worsening of BCVA from 20/40 to 20/80 with keratic precipitation and anterior chamber cells +2. Fundus examination revealed a clear vitreous with normal retinal findings with no evidence of retinal vasculitis or retinal vascular occlusions. Retinal fluid on OCT had completely resolved at that time. A fluorescein angiography (FLA) was performed to rule out any retinal vasculitis or retinal vascular occlusions. The patient was treated with high frequency (initially hourly) administration of prednisolone acetate eye drops (1%) and systemic prednisolone (1 mg/kg body weight) for 3 days and then tapered off over a 2-week period. Further follow-up revealed stable visual acuity (20/60) despite recurrence of IRF. Treatment with brolucizumab was discontinued and the patient was switched back to a previously used anti-VEGF agent with no further adverse events.

Neither patient had a persistent clinically relevant change in visual acuity after IOI was resolved. No occurrence of retinal vasculitis and/or retinal vessel occlusion was observed.

## Discussion

The relatively limited recent experience with intravitreal brolucizumab, as well as the more severe challenge of intraocular treatment-related inflammation, make our data useful and important for further understanding of the role of brolucizumab in the treatment of eyes with refractory nAMD. In this study, we found that brolucizumab appears to be a promising drug for the treatment of such patients, but this must be balanced against a potential risk of adverse events.

Regarding BCVA, our patients achieved statically significant visual improvement at week 16 of treatment, after that, BCVA remained stable compared to baseline until the end of the first year of treatment. This significant visual improvement at week 16 could be due to the intensive therapy with monthly IVIs at the beginning of treatment. This may also suggest that visual outcome could improve by applying IVIs at 4-week intervals after the upload phase. This point was investigated in the MERLIN study. However, upon analysis of the first interpretable data from the MERLIN trial on 28 May 2021, Novartis^®^ discouraged the use of brolucizumab at intervals shorter than 6 weeks after the upload phase because of the disproportionate incidence of treatment-related intraocular inflammation and terminated ongoing studies that allowed for this possibility (Exchange and Koersen, 2021).

On the other hand, the results of our study confirm the efficacy of intravitreal brolucizumab in improving the anatomical outcomes, especially reducing subretinal fluid and pigment epithelial detachment at the end of the first year of treatment and subsequently the mean central macular thickness, which decreased significantly at weeks 4, 28, 36, 44, and 52 of treatment.

There was a statically significant visual improvement at week 16 which was not as evident later, while CMT decreased significantly at the same time point but remained so even afterwards. This phenomenon of discrepancy between anatomy and function has also been reported in other switching studies in patients with nAMD (Gale et al., 2020). This may be explained by the fact that all eyes in our study had a long history of nAMD with chronic IRF, SRF, and PED, resulting in permanent structural changes that could limit the potential of visual improvement despite reduction in CMT.

In addition, our results could be supported by other short-term experiences with brolucizumab (Bulirsch et al., 2021; Montesel et al., 2021; Sharma et al., 2021), which also included previously treated patients and showed significant anatomic improvement on OCT but with no significant visual improvement.

The significant reduction in SRF and PED after 1 year of treatment with brolucizumab in our study are comparable to a similar study from (Book et al., 2021), which also reported similar functional and anatomic results with a significant reduction in SRF and PED after 1 year of treatment.

According to HAWK and HARRIER treatment protocol, the number of injections during the first year of treatment with brolucizumab must range from a minimum of 5 to a maximum of 7 IVIs. Our results showed that patients required 6.4 ± 0.9 IVIs of brolucizumab compared to 9.6 ± 1.9 IVIs of other anti-VEGF agents in the last year before switching. Our results also showed that visual acuity remained stable during this period despite the significantly less number of IVIs, suggesting the conclusion that intravitreal brolucizumab could be able to stabilize visual acuity in patients with refractory nAMD with a significantly less number of IVIs.

This finding be might consistent with the results of the (Haensli et al., 2021) study, which reported that treatment with brolucizumab prolonged the treatment interval in eyes that had responded inadequately to previous anti-VEGF agents. This may play a major role in reducing treatment burdens, which are considered an important cause of non-compliance and undertreatment in many real-world studies (Lad et al., 2014). However, it should be kept in mind that many factors such as the duration of the disease, the type of injury, and most importantly, the prolonged years from therapy with other anti-VEGF agents, may also influence this conclusion.

All of the above benefits of treatment with brolucizumab must be balanced against a potential risk of adverse events, particularly IOI. In this study, we reported two cases of IOI (9.5%) during the first year of treatment. IOI presented as anterior and intermediate uveitis without retinal involvement. However, some papers have reported cases with occlusive vasculitis with severe vision loss after brolucizumab (Haug et al., 2020; Jain et al., 2020).

All anti-VEGF agents may carry the risk of IOI. The overall incidence of sterile IOI after IVI ranges broadly in the literature from 0.005% to 4.4% (Souied et al., 2016; Anderson et al., 2021; Monés et al., 2021). This IOI can range from a mild transient reaction to a potentially sight-threatening outcome. It can manifest as acute onset sterile inflammation or delayed onset inflammatory vasculitis, which has been described with brolucizumab (Anderson et al., 2021; Monés et al., 2021).

A recent study showed a good safety profile as well as lack of inflammatory reactions after multiple bevacizumab IVIs. This was established by evaluating the effects of repeated bevacizumab IVIs on the blood-aqueous barrier (Dolar-Szczasny et al., 2021). A systematic comparison of data from numerous studies of aflibercept, ranibizumab and bevacizumab in nAMD found no difference between their ocular safety profiles, including IOI events (Plyukhova et al., 2020). However, the recent higher incidence of IOI (4.4%) with brolucizumab (Dugel et al., 2021) raises concerns for ophthalmologists treating patients.

The pathogenesis of the IOI events is not yet clear. It may be an auto-immune type IV hypersensitivity reaction (Anderson et al., 2021; Enríquez et al., 2021). It could also be related to the fact that the pharmacological mechanism and pharmacokinetic profile of anti-VEGF agents are different, which in turn affects the risk-benefit ratio (Platania et al., 2015). have already shown that ranibizumab and aflibercept differ significantly both in terms of molecular interactions and stabilizing energy. This calls for further studies on the pharmacokinetic profile of brolucizumab.

Some risk factors have been suggested to play a role in the development of IOI, including:

- Female gender (Witkin et al., 2020; Enríquez et al., 2021), this could be supported by our results, as the 2 IOIs that occurred in our small study were in female patients.
- Patient eyes with history of IOI in the 12 months before the first brolucizumab IVI (Khanani et al., 2021).
- Monthly treatment, most of the IOIs were reported during the first 3 months of treatment (Exchange and Koersen, 2021; Monés et al., 2021).
- Finally, the absence of IOI after 126 brolucizumab IVIs in an Indian study indicates the importance of investigating the potential of race and genetics in predisposing to brolucizumab-related IOI (Chakraborty et al., 2021).

In this study, all patients were followed up 2–5 days after each IVI according to the guidelines of the German Society of Ophthalmology (German Society of Ophthalmology et al., 2021). This is in order to rule out bacterial endophthalmitis, which, unlike noninfectious IOI, typically occurs between days 2 and 5 after IVI. As a consequence, the presence of inflammatory symptoms or signs within the first 5 days after brolucizumab-IVI may be indicative of bacterial endophthalmitis rather than noninfectious IOI. Thus, combined antibiotic and anti-inflammatory therapy might be indicated (Holz et al., 2021). This point was clearly discussed by (Holz et al., 2021). They considered that the prolonged and variable time interval between brolucizumab-IVI and the occurrence of IOI (Baumal et al., 2021) provided no reason to modify the usual approach to IVI follow-up at this point. However, they emphasize the importance of educating patients about the symptoms of IOI and advise them to report immediately if adverse ocular events are noted, even if the IVI was some time ago.

Several experts recommend the suspension of the current brolucizumab treatment with intensive corticosteroid treatment depending on the severity of the inflammation (Baumal et al., 2021; Holz et al., 2021).

Finally, the main potential limitations of our study were the retrospective nature of the work, a relatively small population from a single medical center, and the lack of a control group. Therefore, further studies should be conducted in a much larger population and over a longer follow-up period to provide a more reliable conclusion about the role of brolucizumab in the treatment of eyes with neovascular age-related macular degeneration in our real-world practice.

## Conclusion

In the first year of treatment, intravitreal brolucizumab was able to stabilize visual acuity with significantly less IVIs in patients with refractory macular edema due to nAMD. It also improved anatomic outcomes in these patients, particularly reducing subretinal fluid and pigment epithelial detachment and subsequently central macular thickness. However, two cases of intraocular inflammation were reported as adverse events.

## Data Availability

The raw data supporting the conclusion of this article will be made available by the authors, without undue reservation.
